# Conservative Treatment for Acute Ankle Sprain: A Systematic Review

**DOI:** 10.3390/jcm9103128

**Published:** 2020-09-27

**Authors:** Ana Belen Ortega-Avila, Pablo Cervera-Garvi, Ana Marchena-Rodriguez, Esther Chicharro-Luna, Christopher J. Nester, Chelsea Starbuck, Gabriel Gijon-Nogueron

**Affiliations:** 1 Department of Nursing and Podiatry, Faculty of Health Sciences, University of Malaga, 29071 Malaga, Spain; pcervera@uma.es (P.C.-G.); amarchena@uma.es (A.M.-R.); gagijon@uma.es (G.G.-N.); 2 Biomedical Research Institute (IBIMA), 29010 Malaga, Spain; 3 Department of Behavioral and Health Sciences, Nursing Area, Faculty of Medicine, Miguel Hernández University, San Juan de Alicante, 03550 Alicante, Spain; ec.luna@umh.es; 4 Faculty of Health and Society, University of Salford, Manchester M6 6PU, UK; C.J.Nester@salford.ac.uk (C.J.N.); C.Starbuch@salford.ac.uk (C.S.)

**Keywords:** ankle sprain, conservative treatment, systematic review, pain, function

## Abstract

The aim was to identify conservative treatments available for acute ankle sprain and to evaluate their effectiveness with respect to pain relief and short-term recovery of functional capacity. A systematic review of the relevant literature was conducted via a data search of the PROSPERO, PubMed, Scopus, CINAHL, PyscINFO and SPORTDiscus databases, from inception until December 2019, focusing on randomised control trial studies. Two of the authors independently assessed the quality of each study located and extracted the relevant data. The quality of each paper was assessed using the Cochrane risk of bias tool included in RevMan 5. In all, 20 studies met the inclusion criteria. In terms of absence of bias, only nine papers were classed as “high quality”. Studies (75%) were of low quality in terms of the blinding of participants and personnel and uncertainty in blinding of outcome assessment and all presented one or more other forms of bias. Despite the generally low quality of the studies considered, it can be concluded that conservative treatment for acute ankle sprain normally achieves pain relief and rapidly improved functionality. Research based on higher-quality study designs and procedures would enable more definitive conclusions to be drawn.

## 1. Introduction

Ankle sprain is the most prevalent musculoskeletal injury affecting the lower limb in physically active individuals [1]. It consists of the stretching or partial or complete tearing of one or more ligaments in the ankle joint caused by an involuntary twisting movement that exceeds the normal limits of the joint [2].

The most common mechanism of injury in ankle sprain is the combination of inversion and adduction of the foot in conjunction with plantarflexion (supination), which most usually provokes a deterioration of the external lateral ligament and also often impacts on the anterior peroneal tendons [3]. In exceptional circumstances, the anterior ligament may be torn, with associated capsular damage, and rupture of the peroneal tendons. The deltoid ligament may be damaged by traumatic eversion; although this type of sprain occurs only rarely, the possibility of associated injuries such as distal or proximal fracture of the fibula and even of the talus should be considered [4]. Furthermore, hyperdorsiflexion could damage the syndesmotic ligaments [5]. In addition to ligaments, other anatomic structures such as bone, muscles, tendons, nerves and vascular vessels may be affected [2].

The clinical manifestations of ankle sprain include the inability to walk or even move the joint, a searing or tearing sensation, pain that increases with mobility, colour change and rapid bruising. The intensity of these manifestations depends on the severity of the sprain [6]. Treatments to heal the structures and recover functionality after a sprain may be conservative or surgical. Conservative treatment is usually applied for Grade I and II sprains, and Grade III lesions are treated surgically, although for the latter a conservative approach is sometimes considered sufficient [7].

A wide range of conservative treatments are available, including short-term immobilisation [8], complete immobilisation, ice packs [9], local or systemic non-steroidal anti-inflammatory drugs (NSAIDs) [10], physical therapy [11] and electrical stimulation (with or without muscle contraction) [12]. All have been investigated for efficacy in the resolution or improvement of clinical manifestations of ankle sprain, in areas such as the persistence swelling [13] or the patient’s ability to return to work [14] or to playing sport [15]. However, these reviews have considered situations not only of acute sprain [16,17], but also of chronic ankle instability [18] or a combination of conservative and surgical treatments [19,20]. To our knowledge, none have focused specifically on acute ankle sprain.

In view of these considerations, our study aims to identify conservative treatments for acute ankle sprain and to evaluate their effectiveness in terms of pain relief and rapid recovery of functional capacity.

## 2. Methods

The review protocol was registered at the International Prospective Register of Systematic Reviews (PROSPERO: CRD 42020162500).

### 2.1. Design

This review was performed in accordance with the Preferred Reporting Items for Systematic Reviews and Meta-Analyses (PRISMA) statement [21].

### 2.2. Search Strategy

One member of the research team (ABOA) carried out the search to ensure that no previous studies had been conducted with the same study aim as our own. The following databases were searched: PROSPERO, PubMed, Scopus, CINAHL, PyscINFO and SPORTDiscuss, from inception until December 2019 using optimised search strategies (Appendix A). References were exported and duplicate articles removed using reference management software (Mendeley Desktop v 1.19.4).

### 2.3. Eligibility Criteria

The following eligibility criteria were applied:

- In every case, the study population was diagnosed with acute ankle sprain and given conservative treatment as the first option.
- All studies included in the review were randomised controlled clinical trials (RCTs) in which one or more types of conservative treatment were applied in response to an acute ankle sprain, with a maximum of 7 days after initial injury.
- All the studies included evaluated pain, functionality and/or disability caused by an ankle sprain, using one or more measurement instruments.
- The language of publication was Spanish or English.

Studies of the following types were excluded:

- The study population was diagnosed with chronic or recurrent ankle sprain.
- Those in which both conservative and surgical treatments were applied.
- Those not consisting of an RCT (such as pilot studies, research protocols or quasi-experimental studies).
- Those in which the assessment of risk of bias, using the Cochrane risk of bias tool included in RevMan 5 was high risk (it was not consider random sequence generation, allocation concealment and blinding of participants and personnel).

### 2.4. Study Selection

In the first stage of the review, a double-blinded assessment of titles and abstracts was carried out by two reviewers (P.C-G and A.M-R), working independently, to determine whether each item met the requirements for inclusion. In case of doubt, the full text of the article was evaluated. Disagreements between the two reviewers were resolved by discussion, or if consensus was not possible, a further opinion was sought. It was also planned, if necessary, to send an email to the original authors to obtain further information regarding the study details, but in no case was this measure necessary.

### 2.5. Data Extraction

The following data were extracted from each study, using a standardised template: study details (author; year and country of publication), study participant characteristics (number of patients included in the sample, mean age, sex), characteristics of the sprain, study design, type of conservative treatment administered (intervention group and patients included), follow-up period and measurement instrument used.

No meta-analysis was carried out, due to the heterogeneity of the populations, follow-up characteristics and outcomes included in these studies.

### 2.6. Quality Assessment

Two reviewers (P.C-G and A.M-R), working independently, assessed the risk of bias in the studies considered, using the Cochrane risk of bias tool included in RevMan 5 for this purpose [22]. The following biases were assessed: random sequence generation, allocation concealment, blinding of participants and personnel, blinding of outcome assessment, attrition bias, selective reporting and other bias. Each criterion outcome was classed as high risk, low risk or unclear.

## 3. Results

**Figure 1:**
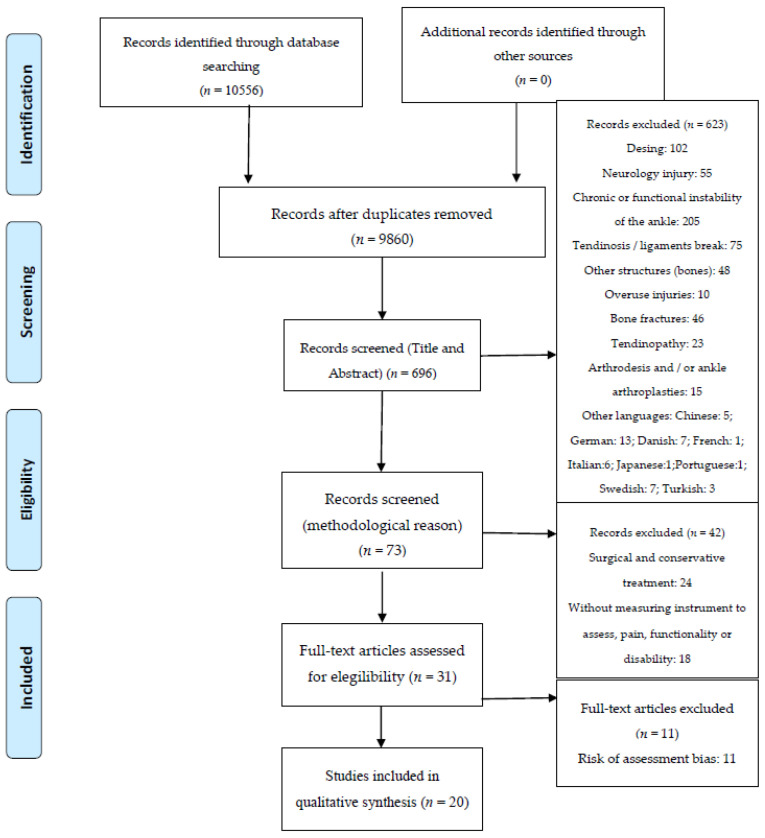
Preferred Reporting Items for Systematic Reviews and Meta-Analyses (PRISMA) Flow Diagram.

An initial 10,556 studies were identified, but 9860 were duplicated among the different databases. The remaining 696 were screened against our inclusion/exclusion criteria, using the titles, abstracts and keywords, resulting in 31 studies that met the inclusion criteria. After quality appraisal (Risk of assessment bias), a further 11 were excluded, and so 20 studies remained in the final qualitative analysis. Figure 1 shows the PRISMA flow diagram for the studies included in the review [23].

### 3.1. Study Characteristics

The studies included a total of 2236 patients with a mean age of 28.86 years. Of these patients, 40.3% were female and 59.7% were male. 

The conservative treatment applied was mainly for acute ankle sprains, Grades I, II or III. In many cases, the location of the sprain (right or left ankle) was not specified. The time elapsed from the start of the injury to the start of conservative treatment was recorded. This time was usually less than 48 h except in two studies which described a period of less than 5 days. The minimum follow-up period recorded was four weeks, with an average of 8.5 weeks (162 days).

The most common treatment described was based on manual or physiotherapeutic methods (eight studies), followed by the use of different types of bandage (three studies) (Table 1). The studies using one or more of the following measurement instruments: Visual Analogue Scale, McGill Pain Questionnaire, Numerical Pain Rating Scale, Total Function Score, Lower Extremity Functional Scale, EuroQol-5D (EQ-5D), American Orthopedics Foot and Ankle Score, Lower Limb Task Questionnaire, Motor Activity Scale, Karlsson Score, Adapted Hughston Clinic Subjective Rating Scale for Ankle disorders, Short Form-12 (SF-12) Foot and Ankle Outcome Score, Foot and Ankle Ability Measure. The most used instrument for evaluating the pain of the ankle sprain is the VAS, used in 13 of the found studies. On another hand, the instruments used more for the evaluation of the function are the SF-12, as a general instrument, and the LEFS as a specific instrument, and both are used in 3 different studies.

The measurement instruments used to assess improvement in terms of pain relief and the recovery of functional capacity in patients with an acute ankle sprain after the application of conservative treatment showed that in most cases significant improvement was achieved (*p* < 0.001) (Table 2). In all the studies is seen an improvement of the pain and the function in the patients. It is seen that this improvement, most of all of the function, is higher in the studies that made the treatments in a bigger period of time.

### 3.2. Risk of Bias

**Figure 2:**
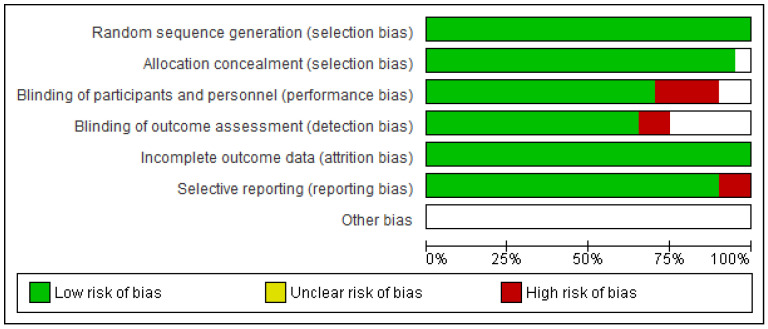
Risk of bias graph.

**Figure 3:**
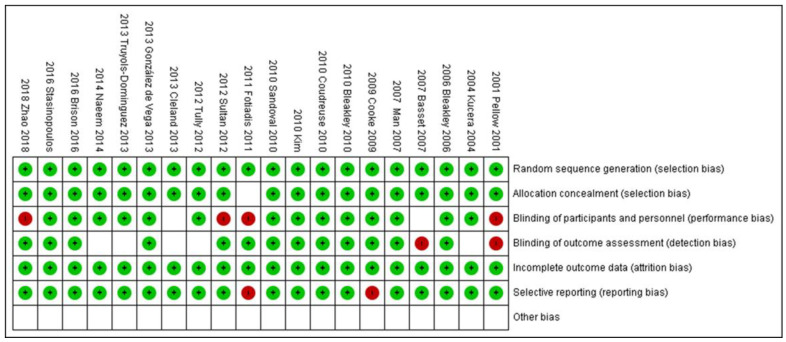
Risk of bias summary.

The risk of bias was evaluated in 20 studies (Figure 2 and Figure 3). Only nine studies presented a low risk of bias. Most studies (75%) were of low quality in terms of the blinding of participants and personnel and uncertainty in blinding of outcome assessment and all presented one or more other forms of bias. The blindness in the evaluation of the results was the bias less specified in the studies, not making it clear if the blindness of the evaluator was made or not.

## 4. Discussion

This review has two main aims: to identify conservative treatments for acute ankle sprain level, Grades I, II and III, and to evaluate the effectiveness of these treatments in terms of pain relief and rapid recovery of functional capacity.

Concerning the first of these aims, our analysis was focused on RCTs investigating different types of short-term conservative treatment for patients with an acute ankle sprain. These treatment options included programmes of physical therapy (at home [11] or supervised by a physical therapist [28]), the prescription of NSAIDs such as diclofenac or traumeel [10], the use of a functional brace (for example, a tubular bandage or aircast brace) [2] or neuromuscular electrical stimulation [32]. On many occasions, these treatments are provided in conjunction with cryotherapy (ice packs) [27] and usual care (consisting of ankle protection, rest, the application of a compression bandage, elevation, analgesics as necessary and a gradual return to weight bearing activities) [9]. In all cases, notable pain relief is obtained and functional capacity regained, during the follow-up period considered, i.e., ranging from seven days to nine months (*p* < 0.001).

Among the studies that focused on identifying treatment effectiveness in terms of pain relief, special attention is paid to the use of cryotherapy, which reduces the sensation of pain when the ice pack is applied intermittently [27], although when it is combined with an exercise intervention programme initiated at an early stage, i.e., after the first week following the occurrence of the sprain, significantly improved results are obtained (*p* < 0.05) after a 16-week follow-up period [28]. In studies that have analysed the recovery of function following the application of conservative treatment, usual care [9], therapeutic physical intervention at home or supervised by a physical therapist [11,28] or the application of bandages are the methods most commonly employed [2]. The results published show there are no significant differences between the different intervention groups in terms of the improvement obtained, after a maximum follow-up period of nine months.

Regarding the presence of bias in the studies considered, our results show that these RCTs are generally of low quality, with only nine studies characterised as high quality (i.e., presenting a low risk of bias) [9,10,27,28,30,32,33,35,36]. The common weaknesses of the RCTs are “Blinding of participants and personnel” and “Uncertainty in blinding of outcome assessment and other bias”. We emphasise the importance of these deficiencies, as the research findings are inherently less reliable if the participants or the researchers are aware of the intervention that has been assigned. In consequence, the results obtained in terms of pain relief and recovery of functionality must be considered invalid and therefore not transferrable, having been altered by the presence of subjectivity and by the patient’s degree of adherence to treatment. Other types of bias may also be present if the procedure applied is not clearly described.

Recent findings indicate that different types of conservative treatment for patients with acute ankle sprain Grades I, II or III produce significant beneficial effects regarding pain relief and the recovery of functionality. However, very few studies of high methodological quality have focused on this study objective. In addition, a wide variety of treatments, measurement tools and follow-up periods have been reported. Our review findings are in line with those of Kosik et al., 2017 [40], Van Ochten et al., 2014 [41] and Kamper et al., 2012 [7]. These reviews, however, examine not only conservative treatment but also surgical methods and their application to patients with chronic ankle instability. Similarly, while Al bimani et al., 2019 [15] assessed the effectiveness of conservative treatments in enabling the patient to return to playing sports, the review takes into account all types of research design. Another of the reviews considered, by Feger et al., 2015 [42], assessed only electrical stimulation or functional treatment [13]. Moreover, the follow-up period considered is only ten weeks. Overall, nevertheless, these reviews highlight the general improvement achieved by patients from the treatments described, although they emphasise the need for further research with appropriate study methods, a common measurement instrument and sufficiently long-term follow-up.

The present systematic review presents numerous strengths. To our knowledge, it is the first to examine only conservative treatments for patients with acute ankle sprains, Grades I, II or III, and in which all studies included are RCTs (performed up to December 2019). Moreover, we applied specific instruments to analyse the risk of bias, and employed a rigorous methodological process, based on a literature search of six medical databases with no time limitation. On the other hand, certain limitations must be acknowledged. The first is the small number of studies extracted that focus on our study objective. In addition, the non-specificity of the location of the sprain (left or right ankle) is unfortunate, as this information could usefully be taken into account to determine whether there is a direct relationship with the laterality of the patient. Another factor is the heterogeneity of the data presented (several measurement instruments were used), which made it impossible to carry out a meta-analysis and, therefore, prevented us from conducting a joint assessment. Only two languages of publication (Spanish or English) were inclusion criteria, which increases the loss of some randomised control trial studies. Finally, there was a relatively high risk of common bias across the studies reviewed.

The most relevant clinical implications are conservative treatments for acute ankle sprain relieve pain and functional capacity, but the results showed there are no significant differences between the different conservative interventions in terms of the improvement obtained, after a maximum follow-up period of nine months. Clinicians should establish a protocol in terms of prevention and thus avoid recurrences or chronic ankle instability.

## 5. Conclusions

Despite the generally low quality of the studies considered, it can be concluded that conservative treatments for acute ankle sprain relieve pain and achieve a rapid return to functionality. However, there is no evidence that any one form of conservative treatment is more effective than any other in terms of these parameters, for patients with acute ankle sprain Grades I, II or III, since a wide range of treatments have been studied for this pathology, using diverse measurement instruments. Future research in this field should ensure homogeneity in the size and composition of the study groups, in the follow-up period and in the description of the main outcomes considered, thus limiting the risk of bias. Research based on higher-quality study designs and procedures would enable more definitive conclusions to be drawn.

## Figures and Tables

**Table 1:** Characteristics of the studies included in the review.

Author Year Country	Patients (n)	Age (years)	Sex		Type of RCT Design	Type of Sprain	Type of Treatment	Follow Up	Outcome
			Female	Male					
Pellow JE et al., 2001 [24]. South Africa	30	15–50 Total mean age: 24.9 Group 1: 23.7 Group 2: 26.1	11 Group 1: 9 Group 2: 2	19 Group 1: 6 Group 2: 13	Single-blind, comparative, controlled study	Subacute ankle inversion sprains (<48 h after initial injury)	Mortise separation adjustment, group 1 (*n* = 15) Detuned ultrasound machine, group 2 (*n* = 15)	28 days	McGill Pain Questionnaire Numerical Pain Rating Scale 101
Kucera et al., 2004 [25]. Prague	203	18–50 Group 1: 27.7 Group 2: 28.3	78 Group 1: 38 Group 2: 40	125 Group 1: 66 Group 2: 59	Randomised, double-blind clinical multicentre parallel study	Acute lateral ankle distortions (24h after the injury)	Verum, Group 1: Cream 10% Reference, Group 2: Cream 1%	14 days	VAS-10
Truyols-Dominguez S. et al., 2013 [26]. Spain	50	28–38 Total mean age: 33	13 Group 1: 6 Group 2: 7	37 Group 1: 19 Group 2: 18	Randomised clinical trial	Acute inversion ankle sprain Grade I and II (Injured <5 days)	Thrust and nonthrust manipulation and exercise interven tion, group 1 (*n* = 25) The same protocol plus myofascial manual therapy techniques, group 2 (*n* = 25)	28 days	Numeric pain rating scale Total Functional Score for Assessment of Acute Lateral Ankle Sprains
Bleakley et al., 2006 [27]. UK	89	Total mean age: 29.9 Group 1: 29.8 Group 2: 31.2	31 Group 1: 15 Group 2: 16	58 Group1: 28 Group2: 30	Randomised controlled trial, double-blind	Mild/moderate ankle sprain <48h after injury) Grades I and II	Intermittent ice, group 1 (*n* = 43) Standard ice application, group 2 (*n* = 46)	42 days	Binskley’s lower extremity functional scale VAS
Cooke et al., 2009 [2]. UK	584	16–72 Total mean age: 30 Group 1: 31 Group 2: 30 Group 3: 29 Group 4: 30	247 Group 1: 64 Group 2: 54 Group 3: 65 Group 4: 64	337 Group 1: 80 Group 2: 88 Group 3: 84 Group 4: 85	Multicentred RCT with blinded assessment of outcome	Acute severe ankle sprain	Group 1: Tubular bandage (*n* = 144) Group 2: Below-knee cast (*n* = 142) Group 3: Aircast brace (*n* = 149) Group 4: Bledsoe boot (*n* = 149)	270 days	FAOS Functional Limitations Profile SF-12 EQ-5D VAS
Bassett et al., 2007 [11]. New Zealand	47	13–62	19 Group 1: 11 Group 2: 8	28 Group 1: 14 Group 2: 14	Controlled trial	Acute ankle sprain (first-time) Grades I, II and III	Clinical intervention, group 1 (*n* = 25) Home intervention, group 2 (*n* = 22)	14 days	Lower Limb Task Questionnaire Motor Activity Scale
Bleakley et al., 2010 [28]. UK	101	16–65	32 Group1: 17 Group2: 15	69 Group1: 34 Group 2: 35	Randomised controlled trial, blinded outcome assessor	Acute ankle sprain Grade I or II	Standard, group 1 (*n* = 51) Exercise, group 2 (*n* = 50)	112 days	VAS LEFS
Brison et al., 2016 [9]. Canada	504	-	280 Group1: 146 Group 2: 134	224 Group 1: 108 Group 2: 116	Randomised controlled trial	Simple Grade I or II ankle sprain.	Physiotherapy, group 1 (*n* = 254) Usual care, group 2 (*n* = 250)	180 days	Foot and Ankle Outcome Score
Cleland et al., 2013 [29]. USA	74	16–60	36 Group 1: 19 Group 2: 17	38 Group 1: 18 Group 2: 20	Randomised clinical trial, non-blinded	Inversion ankle sprain, acute and subacute	Manual therapy and exercise, group 1 (*n* = 37) Home exercise programme, group 2 (*n* = 37)	180 days	FAAM LEFS Numeric pain rating scale
Coudreuse et al., 2010 [30]. France	233	18–65	86	148	Randomised, double-blind, placebo-controlled study	Lateral ankle sprain (<48 hours after the injury)	Novel plaster with diclofenac, epolamine and heparin, group 1 Placebo plaster, group 2	7 days	VAS
Fotiadis et al., 2011 [31]. Greece	79	Mean age Group 1: 38.21 Group 2: 35.35	35 Group 1: 20 Group 2: 15	44 Group 1: 22 Group 2: 24	Prospective randomised study	Type II and III acute (less than 24 h) lateral ankle sprain	Group 1: Micronized purified flavonoid fraction (Daflon 1000 mg) (*n* = 42) Group 2: (Control group) Standard treatment (*n* = 39)	20 days	VAS
Gonzalez de Vega et al., 2013 [10]. Spain	420	18–40	112 Group 1: 39 Group 2: 39 Group3: 34	308 Group 1: 104 Group 2: 101 Group 3: 103	Multicentre, randomised, blinded and active-controlled study	Acute unilateral ankle sprain within the past 24 h. Grades I, II and III	Traumeel ointment (T-O), group 1 (*n* = 143) Traumeel gel (T-G), group 2 (*n* = 140) Diclofenac gel, group 3 (*n* = 137)	42 days	VAS FAAM
Man et al., 2007 [32]. UK	34	Total mean age 30.2 Group 1 34 Group 2 29 Group 3 28	11	23	Randomised trial	Acute ankle sprain injury (within 5 days)	Neuromuscular electrical stimulation treatment, group 1 (*n* = 11) Submotor ES treatment (control group), group 2 (*n* = 11) Sham ES, group 3 (*n* = 12)	14 days	Adapted Hughston Clinic Subjective Rating Scale for Ankle Disorders score
Kim et al., 2017 [33]. South Korea	22	Total mean age: 17.72	0	22	Cross-over randomised design	Grades I and II lateral ankle sprain	Ankle balance taping group 1 Placebo taping group 2 No taping group 3	28 days	VAS
Naeem et al., 2014. [34]. Pakistan	120	Group 1: 28.77 Group 2: 29.83	77 Group 1: 35 Group 2: 42	43 Group 1: 25 Group 2: 18	Level I Randomised controlled trial	Grade I or II lateral ankle sprain	Functional treatment tubigrip, group 1 (*n* = 60) Plaster of Paris, group 2 (*n* = 60)	42 days	VAS Karlsson score
Sandoval et al., 2010 [35]. Colombia	28	Total mean age: 21 Group 1: 21.3 Group 2: 22.5 Group 3: 20.3	10	18	Double-blind, controlled clinical trial	Grade I and II sprain mild or moderate, non-severe	Conventional treatment, group 1 (*n* = 10) HVPC (+) group 2. Conventional treatment and HVPC (positive polarity) (*n* = 8) HVPC (−) group 3. Conventional treatment and HVPC (negative polarity) (*n* = 10)	56 days	VAS
Stasinopoulos et al., 2016 [36]. Greece.	50	18–35 Group 1: 27.92 Group 2: 27.96	15 Group 1: 8 Group 2: 7	35 Group 1: 19 Group 2: 16	Single-centre, parallel group, single-blind, controlled study	Acute ankle sprain Grade II	Group 1: Cryotherapy plus Bioptron light therapy (*n* = 27) Group 2: Control group, cryotherapy only (*n* = 23)	5 days	VAS
Sultan et al., 2012 [37]. England	36	Group 1: 30 Group 2: 34	-	-	Single-centre, randomised, single-blinded, clinical trial	Ankle sprains sustained within 72 h. Grade I, II, III.	Tubigrip, group 1 (*n* = 18) Elastic stocking, group 2 (*n* = 18)	56 days	VAS SF12
Tully et al., 2012 [38]. Northern Ireland.	52	16–65 Group 1: 24.1 Group 2: 26.1 Group 3: 21.9	23 Group 1 6 Group2: 8 Group 3: 9	29 Group 1: 10 Group 2: 10 Group 3: 9	Randomised controlled trial	Acute ankle sprain (<7 days) Grade I or II	Standard, group 1 (*n* = 16) Exercise, group 2 (*n* = 18) Non-injured control. Group 3 (*n* = 18)	7 days	Lower Extremity Functional Scale VAS
Zhao et al., 2018 [39]. China.	62	Group 1: 34 Group 2: 30 Group3: 33	-	-	Randomised controlled trial	Acute ankle sprains identified at 48 hours since the injury. Grades I and II	Standard treatment (RICE), group 1 (*n* = 19) Standard treatment (RICE) plus acupressure therapy, group 2 (*n* = 21) Standard treatment plus mock acupressure therapy, group 3 (*n* = 22)	56 days	VAS American Orthopedic Foot and Ankle Score SF12v2

RCT: Randomised control trial; VAS: Visual Analogue Scale; FAOS: Foot and Ankle Outcome Score; SF-12: Short Form-12; EQ-5D: EuroQol-5D; LEFS: Lower Extremity Functional Scale; FAAM: Foot and Ankle Ability Measure.

**Table 2:** Reported outcomes for pain relief and recovery of functional capacity.

Author	Outcome	Treatment													
Pellow JE et al. [24]		Experimental group: Mortise separation adjustment						Control group: Detuned ultrasound machine							
		Pre	Post 1 month			*p*-value		Pre		Post 1 month			*p*-value		
	McGill Pain Questionnaire	0.20	0.03			0.42		0.24		0.12			0.01		
	NPRS (0–10)	28.73	8.33			0.72		30.73		16.87			0.040		
Kucera et al. [25]		Cream 10%						Cream 1%							
		Visit 3/4	Visit 14					Visit 3/4		Visit 14					
	VAS-10 pain at rest (mm)	28.7 ± 17.1	43.9 ± 22.3 46					14.7 ± 13.5		41.6 ± 21.1					
	VAS-10 functional (mm)	28.7 ± 18.0	50.8 ± 18.9					18.1 ± 13.6		48.1 ± 19.8					
Truyols-Dominguez S. et al. [26]		Experimental Group						Comparison Group							
		Pre-treatment	Post-treatment					Pre-treatment		Post-treatment			Pre *p*-value		
	NPRS (0–10)	5.4 ± 2.0	2.1 ± 1.4					5.1 ± 1.0		3.2 ± 1.5			0.641		
	Total Functional Score	38.9 ± 8.8	78.6 ± 13.9					40.9 ± 18.0		64.0 ± 17.8			0.621		
Bleakley et al. [27]		Intermittent ice group						Standard ice application group					*p*-value		
	LEFS	24.6 ± 1.96						22.3 ± 2.23					0.38		
	Pain intensity at rest (0–10)	1.0 ± 0.16						1.7 ± 0.22					0.08		
	Pain intensity activity (0–10)	3.9 (0.28)						4.7 (0.27)					0.3		
Cooke et al. [2]		Tubular bandage (mean)	Bledsoe (difference)					Aircast difference		Below-knee cast difference					
		4 weeks	9 months	4 weeks		9 months		4 weeks		9 months	4 weeks		9 months		
		Score	Score	Score	ES	Score	ES	Score	ES	Score	ES	Score	ES	Score	ES
	FAOS pain	62.3	81.1	0.6	0.03	1.7	0.09	3.5	0.19	1.9	0.10	5.1	0.28	4.3	0.23
	FAOS symptoms	59.8	79.2	−0.8	−0.04	−1.1	−0.06	2.2	0.12	0.1	0.01	3.8	0.21	0.4	0.02
	FAOS ADL	82.3	93.1	−0.1	−0.01	0.1	0.01	0.6	0.05	1.0	0.10	3.0	0.24	1.2	0.12
	FAOS sports	44.7	76.8	−0.3	−0.01	1.0	0.04	0.0	0.00	0.8	0.03	5.0	0.20	2.4	0.10
	FAOS QoL	43.0	64.9	1.9	0.08	4.0	0.15	4.9	0.22	6.1	0.24	5.9	0.26	6.3	0.24
	FLP ambulatory	16.9	6.3	0.1	0.01	−1.5	−0.18	−0.1	0.00	−2.2	−0.26	−3.1	−0.24	−1.7	−0.21
	SF-12 physical	39.2	49.7	−1.3	−0.16	0.2	0.03	−1.4	−0.17	−0.1	−0.01	2.2	0.27	0.3	0.04
	SF-12 mental	43.4	47.7	1.0	0.10	1.4	0.14	0.1	0.01	1.8	0.18	−0.6	−0.05	1.2	0.12
	EQ-5D	0.60	0.73	0.03	0.14	0.06	0.28	0.00	0.02	0.05	0.25	0.06	0.28	0.04	0.18
	VAS pain at rest	19.2	10.1	−0.7	−0.04	0.7	0.05	−0.7	−0.04	−2.9	−0.19	−4.8	−0.27	−0.8	−0.05
Bassett et al. [11]		Clinical intervention group						Home intervention group							
		Pre		Post				Pre		Post					
	LLTQ recreational subscale	27.92 ± 11.36		12.00 ± 10.10				20.27 ± 12.58		8.18 ± 7.24					
	LLTQ ADL subscale	13.72 ± 11.29		2.32 ± 3.60				7.18 ± 7.06		1.82 ± 3.58					
	Motor Activity Scale	1.20 ± 2.00		5.14 ± 1.28				1.77 ± 1.60		5.73 ± 1.08					
Bleakley et al. [28]		Standard						Exercise							
		Score						Score					*p*-value		
	Pain intensity at rest	1.7 ± 0.22						1.0 ± 0.16					0.008		
	Pain intensity on activity	4.7 ± 0.27						3.9 ± 0.28					0.3		
	Subjective function (LEFS)	22.3 ± 2.23						24.6 ± 1.96					0.38		
Brison et al. [9]		Physiotherapy group						Usual care group							
		1 month	6 months					1 month	6 months	*p*-value 1 month			*p*-value 6 months		
	FAOS	23/180	92/165					33/213	113/174	0.65			0.09		
Cleland et al. [29]		Home Exercise Programme			Manual Therapy and Exercise (MTEX)					Between-Group Differences					
		4 weeks	6 months		4 weeks			6 months		4 weeks			6 months		
	FAAM ADL (0–100%)	9.6	24.6		21.3			30.8		11.7			6.2		
	FAAM sports (0–100%)	13.8	33.5		27.1			40.7		13.3			7.2		
	LEFS (0–80)	5.6	17.3		18.4			25.3		12.8			8.1		
	NPRS (0–10)	−1.5	−3.1		−2.7			−3.6		−1.2			−0.47		
Coudreuse et al. [30]		DHEP group			Placebo group					*p*-value					
		Baseline	7 days		Baseline			7 days		Baseline			7 days		
	VAS pain (0–100)	73.2 ± 1.0			69.3 ± 1.1					*p* = 0.007			*p* < 0.01		
Fotiadis et al. [31]		Daflon group			Control group					*p*-value					
		2 days	20 days		2 days			20 days		2 days			20 days		
	VAS pain (1–10)	2.26 ± 1.86	0.64 ± 1.39		2.0 ± 1.64			0.32 ± 0.57		0.625			0.908		
Gonzalez de Vega et al. [10]		Traumeel ointment			Traumeel gel					Diclofenac gel					
		Pre	Post		Pre			Post		Pre			Post		
	VAS ankle pain	52.6	3.1		53.1			4.1		55.7			3.1		
	FAAM ADL	51.2	41.7		56.0			40.5		51.2			41.7		
	FAAM Sports	18.8	50.0		25.0			50.0		18.8			50.0		
Man et al. [32]		NMES Group			Submotor ES Group					Sham ES Group					
		Session 1	Session 3		Session 1			Session 3		Session 1			Session 3		
	Adapted HCSRSAD	65 (13)	42 (20)		70 (10)			45 (17)		63 (12)			46 (16)		
Kim et al. [33]		Aquatic exercise			Land-based Exercise					Interaction Effect					
		Baseline	4 weeks		Baseline			4 weeks							
	VAS for pain	5.70 (0.36)	0.17 (0.16)		5.66 (0.36)			0.73 (0.16)		*F* = 3.75			*P* = 0.033		
Naeem et al. [34]		Functional Treatment Tubigrip group			Plaster of Paris (POP) group					*p*-value					
		At presentation	At 6 weeks		at presentation			at 6 weeks		At presentation			At 6 weeks		
	VAS	8.40 ± 0.92	3.88 ± 0.85		8.27 ± 0.94			4.97 ± 0.82		0.434			<0.001		
	Karlsson score	21.17 ± 6.31	76.25 ± 10.67		23.67 ± 5.24			70.10 ± 6.35		0.571			<0.001		
Sandoval et al. [35]		Conventional treatment GC			Conventional treatment EEAV (+)				Conventional treatment EEAV (−)				*p* value		
		First	Last		First			Last	First	Last			First	Last	
	VAS at rest	1.0 ± 1.6	0.03 ± 0.09		1.6 ± 2.8			0	0.8 ± 1.8	0			0.75	0.29	
	VAS palpation	5.8 ± 2.9	0.7 ± 0.84		5.6 ± 3.3			0.4 ± 0.6	6.9 ± 1.4	0.91 ± 0.91			0.53	0.41	
Stasinopoulos et al. [36]		Cryotherapy and Bioptron Light group						Cryotherapy only group					*p*-values		
		Pre-treatment	Post-treatment					Before treatment		Post-treatment			Post-treatment		
	VAS pain (0–10)	6.66 (6.89–6.46)	4.46 (4.62–4.30)					6.62 (6.79–6.41) 62.88		5.34 (5.48–5.28)			*p* < 0.0005		
		Stocking group						Tubigrip							
Sultan et al. [37]		Initial	8 weeks					Initial		8 weeks					
	Total SF-12 score	100 (95–105)	119 (118–121)					100 (94–107)		102 (99–107)					
	VAS score	65 (56–73)	5 (0–11)					66 (59–73)		18 (10–26)					
		Standard group						Exercise group					*p* Value		
Tully et al. [38]		At baseline	At 1 week					At baseline		1 week			Baseline	1 week	
	LEFS	35.31 ± 16.56	54.00 ± 12.61					38.22 ± 19.81		61.63 ± 13.05			0.65	0.10	
	VAS Pain at rest	26.5 (23.3)	7.1 ± 7.5					19.6 (17.5)		3.3 ± 4.4			0.33	0.98	
	VAS Pain with activity	53.06 ± 27.7	34.3 ± 22.9					53.3 ± 22.7		25.7 ± 22.1			0.08	0.26	
Zhao et al. [39]		STG group			APG group					Mock APG group					
		Baseline	8 weeks		Baseline			8 weeks		Baseline			8 weeks		
	VAS pain	5.05	0.26		5.05			0.10		4.86			0.41		
	AOFAS	39.53	97.47		38.14			99.04		38.95			96.86		
	Total SF-12 score	107.63	116.21		106.14			119.67		104.95			112.05		

VAS: Visual Analogue Scale; EQ-5D: EuroQol-5D, LLTQ: Lower Limb Task Questionnaire; NPRS: Numerical Pain Rating Scale; SF-12: Short Form-12; FAOS: Foot and Ankle Outcome Score; FAAM: Foot and Ankle Ability Measure; LEFS: Lower Extremity Functional Scale.

## Appendix A

Searching Strategy.

**Table A1:** **PubMed.** Total articles: 656.

1	Ankle
2	Talocrural
3	Talo-crural
4	Talocalcaneal
5	Talo-calcaneal
6	Talofibular
7	Talo-fibular
8	Ligament
9	Lateral Ligament ankle
10	Medial Ligament ankle
11	1 OR 2 OR 3 OR 4 OR 5 OR 6 OR 7 OR 8 OR 9 OR 10
12	Sprain
13	Strain
14	Ankle injury
15	Ankle sprain
16	Inversion sprain
17	Eversion sprain
18	12 OR 13 OR 14 OR 15 OR 16 OR 17
19	11 AND 18
20	Conservative treatment
21	Conservative management
22	Non-surgical treatment
23	CAST
24	Rehabilitation program
25	Myofascial
26	Conservative program
27	Manual Therapy
28	Physiotherapeutic intervention
29	Bandage
30	Plaster
31	Exercise programme
32	Home exercise
33	RICE
34	Taping
35	TENSE
36	Ultrasound
37	20 OR 21 OR 22 OR 23 OR 24 OR 25 OR 26 OR 27 OR 28 OR 29 OR 30 OR 31 OR 32 OR 33 OR 34 OR 35 OR 36
38	19 AND 37

**Table A2:** **CINAHL.** Total articles: 2176.

1	Ankle Sprains
2	Inversion Sprain
3	Eversion Sprain
4	Ankle Injury
5	1 OR 2 OR 3 OR 4
6	Conservative treatment
7	Conservative management
8	Rehabilitation programs
9	Bandage
10	Physiotherapeutic
11	6 OR 7 OR 8 OR 9 OR 10
12	5 AND 11

**Table A3:** **SCOPUS.** Total articles: 597.

1	TITLE-ABS-KEY (Ankle Sprain)
2	TITLE-ABS-KEY (Inversion Sprain)
3	TITLE-ABS-KEY (Eversion Sprain)
4	TITLE-ABS-KEY (Ankle Injury)
5	1 OR 2 OR 3 OR 4
6	TITLE-ABS-KEY (Conservative treatment)
7	TITLE-ABS-KEY (Conservative management)
8	TITLE-ABS-KEY (TENSE)
9	TITLE-ABS-KEY (Bandage)
10	TITLE-ABS-KEY (Rehabilitation program)
11	TITLE-ABS-KEY (Physiotherapeutic)
12	6 OR 7 OR 8 OR 9 OR 10 OR 11
13	5 AND 12

**Table A4:** **SPORTSDiscus via EBSCOHost.** Total articles: 5618.

1	Ankle Sprains
2	Inversion Sprain
3	Eversion Sprain
4	Ankle injury
5	1 OR 2 OR 3 OR 4
6	Conservative treatment
7	Conservative management
8	Rehabilitation program
9	Physiotherapeutic
10	6 OR 7 OR 8 OR 9 OR 10
11	5 AND 10

**Table A5:** **PsycINFO.** Total articles: 1371.

1	Ankle Sprains
2	Inversion Ankle sprain
3	Eversion Ankle sprain
4	1 OR 2 OR 3
5	Conservative treatment
6	Conservative management
7	Rehabilitation program
8	Physiotherapeutic
9	5 OR 6 OR 7 OR 8
10	4 AND 9

**Table A6:** **PROSPERO.** Total articles: 38.

1	Ankle Sprains
